# Microbial production of sabinene—a new terpene-based precursor of advanced biofuel

**DOI:** 10.1186/1475-2859-13-20

**Published:** 2014-02-10

**Authors:** Haibo Zhang, Qiang Liu, Yujin Cao, Xinjun Feng, Yanning Zheng, Huibin Zou, Hui Liu, Jianming Yang, Mo Xian

**Affiliations:** 1 CAS Key Laboratory of Biobased Materials, Qingdao Institute of Bioenergy and Bioprocess Technology, Chinese Academy of Sciences, No.189 Songling Road, Qingdao, Laoshan District 266101, China; 2 College of Food Science, Sichuan Agricultural University, Yaan 625014, China

**Keywords:** Sabinene, Geranyl diphosphate synthase, Sabinene synthase, *Escherichia coli*

## Abstract

**Background:**

Sabinene, one kind of monoterpene, accumulated limitedly in natural organisms, is being explored as a potential component for the next generation of aircraft fuels. And demand for advanced fuels impels us to develop biosynthetic routes for the production of sabinene from renewable sugar.

**Results:**

In this study, sabinene was significantly produced by assembling a biosynthetic pathway using the methylerythritol 4-phosphate (MEP) or heterologous mevalonate (MVA) pathway combining the GPP and sabinene synthase genes in an engineered *Escherichia coli* strain. Subsequently, the culture medium and process conditions were optimized to enhance sabinene production with a maximum titer of 82.18 mg/L. Finally, the fed-batch fermentation of sabinene was evaluated using the optimized culture medium and process conditions, which reached a maximum concentration of 2.65 g/L with an average productivity of 0.018 g h^-1^ g^-1^ dry cells, and the conversion efficiency of glycerol to sabinene (gram to gram) reached 3.49%.

**Conclusions:**

This is the first report of microbial synthesis of sabinene using an engineered *E. coli* strain with the renewable carbon source as feedstock. Therefore, a green and sustainable production strategy has been established for sabinene.

## Background

Progresses in metabolic engineering and synthetic biology boost the engineering of microbes to produce advanced biofuels [1-3]. Among the bio-based fuels, terpenes, which are derived from the head-to-tail condensation of dimethylallyl pyrophosphate (DMAPP) and isopentenyl pyrophosphate (IPP), and traditionally used in flavorings, fragrances [4], medicines and fine chemicals [5,6], have the potentials to serve as advanced biofuel precursors [7-9].

**Figure 1:**
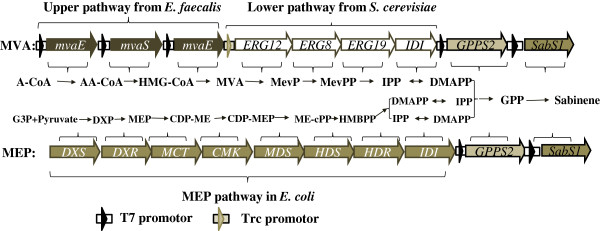
**Sabinene biosynthesis pathway.** Gene symbols and the enzymes they encode (all genes marked with black arrows were from *E. faecalis*, all genes marked with white arrows were isolated from *S. cerevisiae*, the gene marked with gray arrows and black characters were derived from *A. grandis* or *S. pomifera*, and the gene marked with gray arrows and white characters were native genes in *E. coli*). Enzymes in MVA pathway: MvaE, acetyl-CoA acetyltransferase/HMG-CoA reductase; MvaS, HMG-CoA synthase; ERG12, mevalonate kinase; ERG8, phosphomevalonate kinase; ERG19, mevalonate pyrophosphate decarboxylase; IDI, IPP isomerase; *A. grandis* geranyl diphosphate synthase (GPPS2) and *S. pomifera* sabinene synthase (SabS1) were optimized to the preferred codon usage of *E. coli*. Enzymes in MEP pathway: DXS, DXP synthase; DXR, DXP reductoisomerase; MCT, CDP-ME synthase; CMK, CDP-ME kinase; MDS, ME-cPP synthase; HDS, HMBPP synthase: HDR, HMBPP reductase; IDI, IPP isomerase. Intermediates in MVA pathway: A-CoA, acetyl-CoA; AA-CoA, acetoacetyl-CoA; HMG-CoA, hydroxymethylglutaryl-CoA; Mev-P, mevalonate 5-phosphate; Mev-PP, mevalonate pyrophosphate. IPP, isopentenyl pyrophosphate; DMAPP, dimethylallyl pyrophosphate; GPP, geranyl diphosphate. Intermediates in MEP pathway: G3P, glyceraldehyde 3-phosphate; DXP, 1-deoxy-D-xylulose 5-phosphate; MEP, 2-C-methyl-D-erythritol 4-phosphate; CDP-ME, 4-(cytidine-5′-diphospho)-2-C-methyl-D-erythritol; CDP-MEP, 2-phospho-4-(cytidine-5′-di-phospho)-2-C-methyl-D-erythritol; ME-cPP, 2-C-methyl-D-erythritol 2,4-cyclodiphosphate; HMBPP, 4-hydroxy-3-methylbut-2-enyl diphosphate; IPP, isopentenyl diphosphate; DMAPP, dimethylallyl diphosphate.

Terpenes are a large and diverse class of organic compounds, which are mainly produced by a variety of plants. They are generated from the common precursors, IPP and DMAPP, which can be produced from the methylerythritol 4-phosphate (MEP) pathway or the mevalonate (MVA) pathway (Figure 1) [10]. Although many microorganisms harbor the MEP pathway or MVA pathway to supply the intermediates DMAPP and IPP, they are unable to produce the monoterpenes for the lack of monoterpenes synthases. With the rising demand for advanced fuels, terpene-based advance fuels attract more attentions. Many researchers explored microbial methods of monoterpene productions by introducing heterologous monoterpene synthase, including 3-carene, limonene, pinene and bisabolene. Reiling *et al.* engineered *E. coli* strain with overexpression native 1-deoxy-D-xylulose-5-phosphate synthase (DXS), farnesyl diphosphate synthase (IspA), IPP isomerase (IPIHp) from *Haematococcus pluvialis*, and 3-carene cyclase from *Picea abies*, which can accumulate a 3-carene titer of about 3 μg/L/OD_600_ after 8 h production [11]. Carter *et al.* constructed a monoterpene biosynthesis pathway in *E. coli* with a titer of about 5 mg/L limonene production using the native MEP pathway [12]. Bisabolene, α-pinene et al. had been produced using MVA heterologous pathway in microorganisms [9,13,14].

Sabinene (CAS: 3387-41-5), a perfume additive, is being explored as the components for the next generation aircraft fuel [7,8]. Meanwhile, sabinene contributes to the spiciness of black pepper, is a principal component of carrot seed oil, and occurs at a low concentration in tea tree oil. Currently, sabinene is extracted from plants, which is inefficient and requires substantial expenditure of natural resources because of the low content of them [15]. Though sabinene was found in the culture of an endophytic *Phomopsis* sp. as a component of its volatile organic compounds, further work need to be done for microbial production method because of the low tilter in the mixture [16]. Consequently, green and sustainable microbial technologies, which could engineer microorganisms to convert renewable resources from biomass to biobased advanced biofuels, provided an alternative strategy [17-19].

In this paper, sabinene was significantly produced by assembling a biosynthetic pathway using the MEP or heterologous MVA pathway combining the GPP and sabinene synthase genes in an engineered *E. coli* strain. Subsequently, the culture medium and process conditions were optimized to enhance sabinene production. Finally, fed-batch fermentation of sabinene was evaluated using the optimized culture medium and process conditions.

## Results and discussion

### Characterization of sabinene by GC-MS

**Figure 2:**
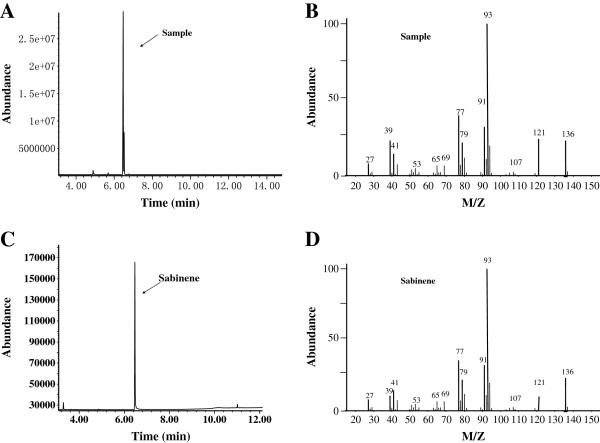
**GC-MS analysis of sabinene from the headspace of the sealed cultures of strain HB2.** Cultures were induced at 37°C, OD_600_ = 0.6-0.9, and final concentration of 0.25 mM IPTG. By comparing with the authoritative sabinene **(A, B)**, the capacities of sabinene biosynthesis were verified. **A**, **C**, total ion chromatogram (TIC); **C**, **D**, mass spectrum. Based on the relative retention time and total ion mass spectral comparison with an external standard, sabinene production was identified.

*E. coli* cannot produce sabinene because of the absence of sabinene synthase, though it possesses a native MEP pathway which can supply the intermediates DMAPP and IPP (Figure 1). Consequently, sabinene synthase (SabS1) derived from *Salvia pomifera* was introduced into the *E. coli* strain (HB1), to synthesize sabinene. However, after 36 h of incubation of the modified strain, only trace of the target product could be detected by GC-MS (data not shown), based on the relative retention time and total ion mass spectral comparison with the external standard. The main reason might lie in the insufficiency of GPP in the host, because the wild *E. coli* seldom produces terpene. Hence, the native gene *IspA* from *E. coli* W3100, which encodes farnesyl diphosphate synthase, was added to enhance the metabolic flux into GPP by catalyzing the conversion of DMAPP and IPP. The gene *IspA* combining with the sabinene synthase gene (*SabS1*) was ligated into pACYCDuet-1 to create the plasmid pHB3 (pACY-*IspA-SabS1*). The *E. coli* strain harboring pHB3 was inoculated in the initial fermentation medium and incubated at 37°C with shaking at 180 rpm in shake-flasks. IPTG was added to a final concentration of 0.5 mM when its OD_600_ reached 0.6-0.9, and culture was further maintained at 37°C for 24 h. The off-gas from the headspace of the sealed cultures was tested by GC-MS. The engineered *E. coli* BL21(DE3) strain harboring the native *IspA* gene and *SabS1* from *S. pomifera* produced sabinene in detectable quantities (shown in Figure 2). Thus, using the MEP pathway and *SabS1* from *S. pomifera*, the biosynthetic pathway for sabinene production was successfully constructed in *E. coli* BL21(DE3). The result also indicated that introduction of GPP synthase was beneficial to enhance the metabolic flux into GPP which would improve the sabinene products efficiently.

### Screening of GPP synthases

GPP synthase, is one of the rate-limiting enzyme in the sabinene synthesis of *E. coli* BL21(DE3) [20]. An effective method to optimize pathway efficiency may be to use genes of rate-limiting enzymes from different organisms [21]. In this study, GPPS enzymes from *Abies grandis* (GPPS2) and *E. coli* were evaluated to enhance the supply of GPP.

**Figure 3:**
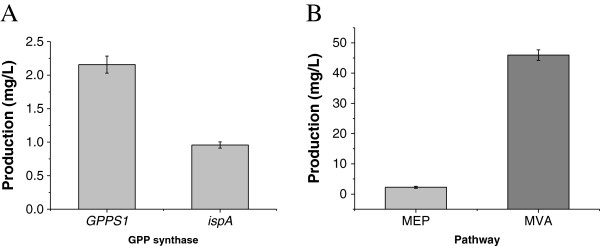
**Effect of GPP synthase and metabolic pathway on sabinene production. A**: Effect of GPP synthase. GPPS1, GPP synthase form *A. grandis*; IspA, GPP synthase from *E. coli*. **B**: Effect of metabolic pathway on sabinene production, the pathway details were described in section Figure 1. The strain harboring GPPS2 can produce 2.2-fold higher concentration of sabinene than IspA, while the strain harboring MVA pathway can produce 20-fold higher concentration of sabinene than MEP pathway.

The *GPPS2* gene from *A. grandis* or *IspA* gene from *E. coli* was cloned into the plasmid pACYCDuet-1 along with the sabinene synthase gene (*SabS1*) to create the plasmid pHB3 or pHB5, respectively, which were subsequently harbored by *E. coli* BL21(DE3) to screen the GPP synthase, because of the difficulty in detecting and quantifying GPP. The strains HB2 (harboring pHB3) and HB3 (harboring pHB5) were cultured in 600-ml shake-flasks with 100 ml of fermentation medium. When each culture reached an OD_600_ of 0.6-0.9, expression of GPP synthase and sabinene synthase was induced by 0.25 mM IPTG, and the culture was further incubated at 37°C for 24 h. A noticeable difference in sabinene production was observed between the two strains. The strain HB3 produced 2.07 mg/L sabinene, while the strain HB2 produced 0.96 mg/L (Figure 3A). This result demonstrates that the exogenous expression of GPPS contributed to the sabinene production, and the enzyme activity of GPPS2 from *A. grandis* was higher than that of IspA from *E. coli* W3100. IspA could give substantial amounts of the larger prenyl diphosphates, FPP and GGPP, in addition to GPP [22], that was why GPPS2 from *A. grandis* was more efficient than IspA in the synthesis of GPP. Hence, the GPPS2 was selected to enhance GPP production in the following experiments.

### Screening of synthetic pathways for sabinene production

The hybrid exogenous MVA pathway is effective to synthesize DMAPP and IPP according to previous experimental data. The recombinant strain HB4 (*E. coli* harboring the MVA pathway, GPPS and sabinene synthase) and strain HB3 (*E. coli* harboring the native MEP pathway, GPPS synthase and sabinene synthase) were cultured to test the effect of the MVA pathway on the production of sabinene, in fermentation medium under shake-flask conditions. The sabinene titer of strain HB4 reached 44.74 mg/L after being induced by 0.25 mM IPTG for 24 h with glycerol as carbon source and beef powder as nitrogen source (Figure 3B). The titer was about 20-fold higher than that of the strain HB3 cultured at the same conditions.

These results indicated that the hybrid MVA pathway caused a huge increase in sabinene production, which was accordant with the production of other terpenes using a hybrid exogenous MVA pathway in engineered *E. coli* strains [23]. One reason for the inefficiency of MEP approach was the regulatory mechanisms present in the native host [24]. This limitation was also confirmed by experiments on isoprene production using the MEP or MVA pathway [23,25]. It is because the hybrid exogenous MVA pathway is effective to synthesize DMAPP and IPP, which are the precursors of GPP. Consequently, we hypothesized that the engineered strain with the hybrid exogenous MVA pathway could further enhance the production of sabinene. Therefore, the strain HB4 harboring the hybrid exogenous MVA pathway was chosen for further experiments.

### Optimization of fermentation medium and culture conditions

Fermentation medium and culture conditions play a vital role in the formation, concentration and yield of the end product [26], and they also provide data for fed-batch fermentation. Optimizing fermentation medium and culture conditions for strains can make the fed-batch fermentation easy to get higher quality and quantity products. In this study, the one-factor-at-a-time method, which is based on the classical method of changing one independent variable while fixing all others [27,28], is applied to optimize medium components as well as process conditions. The four most important factors, carbon source, organic nitrogen source, induction temperature, and inducer concentration were optimized to improve sabinene production, using the strain HB4.

#### Effect of organic nitrogen source on sabinene production

**Figure 4:**
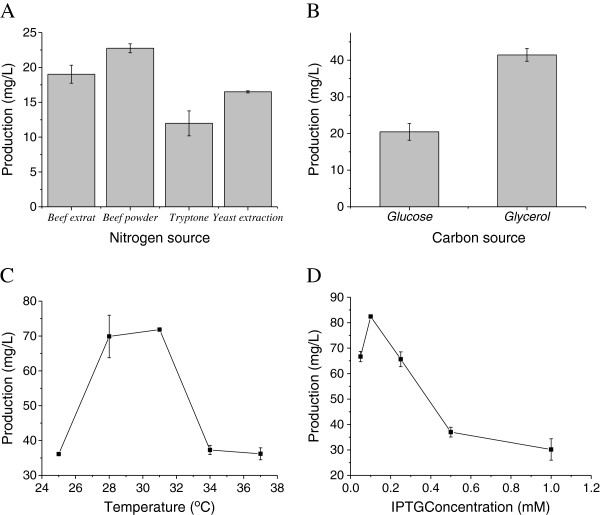
**Effects of fermentation source and culture conditions on sabinene production by HB4. A**: Effect of nitrogen sources on sabinene production; **B**: Effect of carbon sources on sabinene production; **C**: Effect of temperatures on sabinene production; **D**: Effect of the concentration of inducer on sabinene production. When OD_600_ reached 0.6-0.9, cultures were induced for 24 h using IPTG in shake-flasks. All the experiments were performed in triplicates. Optimized conditions: Nitrogen sources, beef power; Carbon source, glycerol; Temperature, 31°C; IPTC concentration, 0.1 mM.

The source of the nitrogen in the medium, especially the organic, which can also provide trace nutrition for micro-being, plays an important role in improving the biosynthesis of desired products [29]. Four different organic nitrogen sources were assessed to investigate the effect of organic nitrogen source on sabinene production (Figure 4A). Beef powder permitted a little higher sabinene production than other organic nitrogen sources, among the organic nitrogen supplements tried. The highest concentration of sabinene was 23.20 mg/L, which was about 1.4 times as much as the lowest observed yeast extract.

#### Effect of carbon source on sabinene production

The source of carbon is the main feedstock in most fermentation media; therefore, finding efficient and cheap carbon source for sabinene production is important. In this study, mostly used carbon source glucose and glycerol were applied to investigate the effect of carbon source on sabinene production. As is shown in Figure 4B, the glycerol permitted a little higher sabinene production than glucose. The highest concentration of sabinene was 41.45 mg/L, which was about 2.03 times as much as that of glucose as carbon source.

#### Effect of induction temperature on sabinene production

In this study, the induction temperatures of 25°C, 28°C, 31°C, 34°C and 37°C were tried to increase sabinene production. As is shown in Figure 4C, the maximum sabinene production was observed at 31°C, at 71.50 mg/L with beef power as nitrogen source and glycerol as carbon source. It was about 2 times greater than that observed at 25°C (36.12 mg/L), and 37°C (36.19 mg/L). The enzyme expression, cell growth and product formation should be balanced in a successful control of cultivation temperature. Because low temperatures decrease the inclusion bodies in genetically engineered *E. coli*, the activities of recombinant enzymes can be enhanced by low induction temperatures (25°C or 30°C) [30,31]. Hence, the optimal induction temperature for sabinene production was around 31°C.

#### Effect of inducer concentration on sabinene production

To optimize the inducer concentration, various IPTG concentrations, ranging from 0.05 mM to 1 mM, were tested. The production of sabinene reached a maximum of 82.18 mg/L at the IPTG concentration of 0.1 mM, which was about 2.45 times greater than those observed at 1.0 mM (33.14 mg/L). The level of IPTG used can be varied to adjust the extent of the metabolic burden imposed on the cell [32], which can result in reduced growth rates, cell yields, protein expression, and plasmid stability [33,34].

Therefore, the most suitable medium was using beef powder and glycerol as the nitrogen and carbon source, respectively, and optimal culture temperature for sabinene production was 31°C at the concentration of 0.1 mM IPTG using the engineered strain HB4.

#### Toxicity of commercial sabinene to *E. coli*

**Figure 5:**
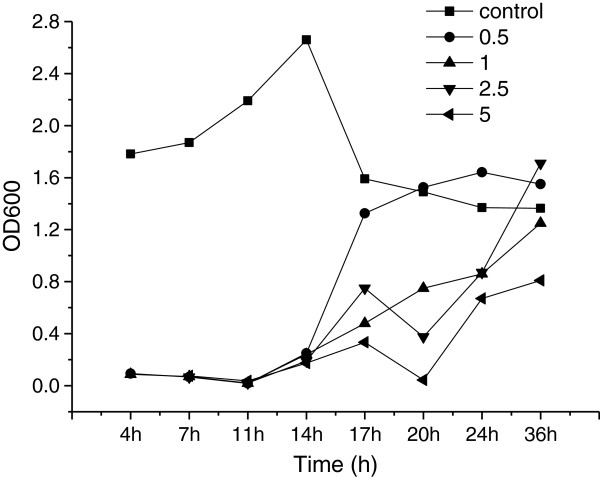
**The growth of *E. coli *in LB medium with different concentration of commercial sabinene.** The growth of the bacterial culture was determined by measuring the OD_600_ (the optical density at 600 nm) with a spectrophotometer (Cary 50 UV-Vis, Varian) at 4 h, 7 h, 11 h, 14 h, 17 h, 20 h, 24 h and 36 h. The concentrations of sabinene were added to the LB medium as follows: 0 g/L (■), 0.5 g/L (●), 1 g/L (▲), 2.5 g/L (▼), 5 g/L (◄).

Toxicity of sabinene to the overproducing organism plays an important role in the biosynthetic process. The commercial sabinene imparts toxicity to *E. coli* when added exogenously to the medium (Figure 5). The *E. coli* cell growth was comparable at 0 g/L and up to 5 g/L of exogenously added sabinene. Though the log phase of *E. coli* was prolonged with about 12 h by even 0.5 g/L sabinene compared with the control, it can grow in 5 g/L sabinene with inhibiting rates of 70% after inoculation and cultured for 36 hours (Figure 5). The results indicated that sabinene can be produced with the engineered *E. coli*, but the production tolerance cannot be neglected to get high titers.

The toxicity of products to the hosts is common in biosynthesis of biofuels and chemicals [3,35]. Expression of efflux pumps, heat shock proteins, membrane modifying proteins, and activation of general stress response genes all can improve tolerance of the hosts [2,3,36]. Furthermore, *in situ* product removal and membrane technology both can be used in the production of sabinene to get high titers [37].

### Fed-batch culture of the engineered strains

**Figure 6:**
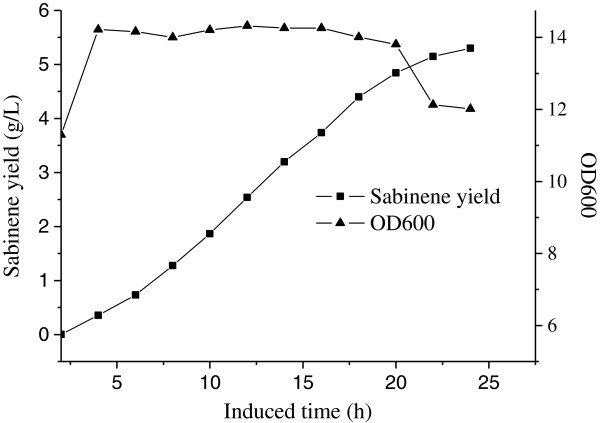
**The time course of sabinene production by HB4 harboring pHB7 and pTrcLower in fed-batch fermentation.** sabinene accumulation (■) and cell growth (▲). Induction was carried out at 12 h at the OD_600_ of 11. The maximum concentration of sabinene was 2.65 g/L with an average productivity of 0.018 g h^-1^ g^-1^ dry cells, and the conversion efficiency of glycerol to sabinene (gram to gram) was 3.49%.

Fed-batch fermentation was carried out using the engineered *E. coli* BL21(DE3) strain simultaneously harboring plasmids pHB7 and pTrcLower, in the optimized medium and culture conditions, to further determine the ability of the engineered strain to produce sabinene at high yield. Glycerol was added continuously when the initial carbon source was exhausted which was indicated by the sharp rise of DO. As is shown in Figure 6, sabinene production increased rapidly from 4 h to 20 h after induction. After the cultures were induced for 24 h, sabinene reached a maximum concentration of 2.65 g/L with an average productivity of 0.018 g /h/g dry cells, and the conversion efficiency of glycerol to sabinene (gram to gram) reached 3.49%.

The maximum cell density of the engineered strain reached only about 14, four hours after being induced with IPTG, with a sabinene titer of no more than 0.5 g/L, which was rather low for the fed-batch fermentation of *E. coli* strains. The main reason for the low cell mass of *E. coli* strain may lie in the retardation of cell growth resulting from toxicity of the product, which was proved by the experiment of toxicity. Meanwhile, overexpression of many heterologous genes may be another reason. To resolve the above-mentioned problems, many possible improvements can be achieved to enhance sabinene production. One approach is to optimize the fermentation process by increasing cell density to elevate the yield of products [38,39], using *in situ* product removal, membrane technology or dissociation of growth or cell mass formation from product formation to reduce the toxicity of sabinene [37]. Another approach is engineering of the host including: employing a chromosome integration technique to decrease the cell growth burden on the host that results from overexpression of heterologous genes [9], expression of efflux pumps, heat shock proteins, membrane modifying proteins, and activation of general stress response genes to improve tolerance of the host to sabinene [2,3,36].

## Conclusions

In this study, sabinene was significantly produced by assembling a biosynthetic pathway using the MEP or heterologous MVA pathway combining the GPP and sabinene synthase genes in an engineered *E. coli* strain. Subsequently, the culture medium and process conditions were optimized to enhance sabinene production. Finally, we also evaluated the fed-batch fermentation of sabinene using the optimized culture medium and process conditions, sabinene reached a maximum concentration of 2.65 g/L with an average productivity of 0.018 g/h/g dry cells, and the conversion efficiency of glycerol to sabinene (gram to gram) reached 3.49%. As far as we know, this is the first report of biosynthesis of sabinene using an engineered *E. coli* strain with the renewable carbon source as feedstock. Therefore, a green and sustainable production strategy has been provided for sabinene from renewable sources in *E. coli*.

## Methods and materials

### Plasmids, bacterial strains, and growth conditions

All plasmids and strains used in this study are listed in Table 1. *E. coli* BL21(DE3) (Invitrogen, Carlsbad, CA) was used as the host to overexpress proteins and produce sabinene. Cultures were grown aerobically at 37°C in Luria Broth (tryptone 10 g/L, NaCl 10 g/L, and yeast extract 5 g/L at pH 7.0-7.4). For initial production of sabinene experiments in shake-flasks, strains were grown in a medium (initial production medium) [40] consisting of the following: 20 g/L glucose, 9.8 g/L K_2_HPO_4_, 5 g/L beef extract, 0.3 g/L ferric ammonium citrate, 2.1 g/L citric acid monohydrate, 0.06 g/L MgSO_4_ and 1 ml/L of trace element solution, which included (NH_4_)_6_Mo_7_O_24_·4H_2_O 0.37 g/L, ZnSO_4_·7H_2_O 0.29 g/L, H_3_BO_4_ 2.47 g/L, CuSO_4_·5H_2_O 0.25 g/L, and MnCl_2_·4H_2_O 1.58 g/L. Ampicillin (Amp, 100 μg/mL) and chloramphenicol (Cm, 34 μg/mL) was added if necessary.

**Table 1:** Plasmids and strains used in this study

**Name**	**Relevant characteristics**	**References**
**Plasmids**		
pACYCDuet-1	P15A origin; Cm^R^; P_T7_	Novagen
pTrcHis2B	ColE1 origin; Amp^R^; P_trc_	Invitrogen
pGH	pUC origin; Amp^R^; P_T7_	Generay
pTrcLower	ColE1 origin; Amp^R^; P_trc_:: *ERG12*-*ERG8*-*ERG19*-*IDI1*	[42]
pHB1	P15A origin; Cm^R^; P_T7_:: *SabS1*	This work
pHB2	P15A origin; Cm^R^; P_T7_::*IspA*	This work
pHB3	P15A origin; Cm^R^; P_T7_::*IspA*-*SabS1*	This work
pHB4	P15A origin; Cm^R^; P_T7_:: *GPPS2*	This work
pHB5	P15A origin; Cm^R^; P_T7_:: *GPPS2-SabS1*	This work
pHB6	P15A origin; Cm^R^; P_T7_:: *mvaE*-*GPPS2*-*SabS1*	
pHB7	P15A origin; Cm^R^; P_T7_::*mvaE*-*mvaS-GPPS2-SabS1*	This work
**Strains**		
*E. coli* BL21(DE3)	*E. coli B dcm ompT hsdS*(rB ^-^ mB ^-^) *gal*	Takara
*E. coli* DH5α	*deoR*, recA1, endA1, hsdR17(rk^-^, mk^+^), phoA, supE44, λ-, thi^-^1, gyrA96, relA1	Invitrogen
*Saccharomyces cerevisiae*	Type strain	ATCC 204508
HB1	*E. coli* BL21(DE3) harboring pHB1	This work
HB2	*E. coli* BL21(DE3) harboring pHB3	This work
HB3	*E. coli* BL21(DE3) harboring pHB5	This work
HB4	*E. coli* BL21(DE3) harboring pHB7 and pTrcLower	This work

Biosensor equipped with glucose oxidase membrane electrodes (Shandong Academy of Sciences, Jinan, China) was applied to determine the concentration of glucose.

### Plasmid construction

The experiments were carried out according to standard protocols [41]. Polymerase chain reaction (PCR) was performed using *Pfu* DNA polymerase (TaKaRa, Dalian, China) according to the manufacturer’s instructions.

### Construction of plasmids for GPP synthase screening

*E. coli* BL21(DE3) genomic DNA was amplified as a template to obtain the *IspA* gene by PCR using the primers IspA-F and IspA-R (Table 2). The *IspA* gene fragment was digested using *Bgl* II and *Nde* I, and subsequently cloned into the corresponding sites of the vector pACYCDuet-1 to create pHB2 (Table 1). The *SabS1* gene fragment (mentioned blow) was obtained by digestion of pGH/Pt30 with *Bgl* II and *Xho* I and was introduced into the corresponding sites of pHB2 to create pHB3.

**Table 2:** Primers used in this study

**Name**	**Sequence (5′ → 3′)**
IspA-F	GGGAATTCCATATGATGGACTTTCCGCAGCAACTC
IspA-R	GGAAGATCTTTATTTATTACGCTGGATGATGT
mvaE-F	CATGCCATGGAGGAGGTAAAAAAACATGAAAACAGTAGTTATTATTGATGC
mvaE-R	CGCGGATCCTTATTGTTTTCTTAAATCATTTAAAATAGCGCGGA TCCTTATTGTTTTCTTAAATCATTTAAAATAG
mvaS-F	CCAGAGCTCAGGAGGTAAAAAAACATGACAATTGGGATTGATAAAATTA
mvaS-R	CAACTGCAGTTAGTTTCGATAAGAGCGAACG

The geranyl diphosphate synthase gene (*GPPS2*, GenBank No. AF513112) from *Abies grandis* and sabinene synthase gene (*SabS1*, GenBank No. ABH07678.1) from *Salvia pomifera* were analyzed by online software (http://www.genscript.com/cgi-bin/tools/rare_codon_analysis) and optimized to the preferred codon usage of *E. coli* (http://www.jcat.de/). The codon-optimized *GPPS2* gene and *SabS1* gene were synthesized by Generay Company with plasmid pGH as the vector (pGH-*GPPS2* and pGH-*SabS1*). The *SabS1* gene fragment was obtained by digestion of pGH-*SabS1* with *Bgl II and Xho I* and then cloned into the corresponding sites of pACYCDuet-1 to create pHB1.The *GPPS2* gene fragment was obtained by digestion of pGH-*GPPS2* with *Nde* I and *Bgl* II and then cloned into the corresponding sites of pACYCDuet-1 to create pHB4. The *SabS1* gene fragment was obtained by digesting pGH-*SabS1* with *Bgl* II and *Xho* I and was ligated into the corresponding sites of pHB4 to construct pHB5.

### Construction of plasmids for the whole pathway of sabinene synthesis

As mentioned above, *E. coli* BL21(DE3) has its native MEP pathway to form IPP and DMAPP. Therefore, the MEP pathway for sabinene synthesis was constructed by harboring the plasmid pHB1 to introduce the exogenous sabinene synthase. Furthermore, to enhance the metabolic flux into GPP by catalyzing the conversion of DMAPP and IPP, the GPP synthase (IspA or GPPS2) was overexpressed or introduced.

*E. coli* BL21(DE3) harboring pHB7 and pTrcLower was constructed to form the MVA pathway for sabinene synthesis. The mvaE (Genbank: AF290092) was amplified with the primer mvaE-F and mvaE-R from genomic DNA of *Enterococcus faecalis* (ATCC 700802D-5) and then cloned into pHB5 and with restriction enzymes *Nco* I and *Bam* HI, creating pHB6. The mvaS (Genbank: AF290092) was amplified from genomic DNA of *E. faecalis* (ATCC 700802D-5) with the primer mvaS-F and mvaS-R and cloned into pHB6 and with restriction enzymes *Sac* I and *Pst* I, creating pHB7. The *ERG12*, *ERG8*, *ERG19* and *IDI1* genes from *S. cerevisiae* (ATCC 204508) were cloned into pTrcHis2B (Invitrogen, Carlsbad, CA) using a method of successive hybridization to yield pTrcLower [42].

### Characterization of sabinene by GC-MS

The *E. coli* strain was inoculated in 50 ml of fermentation medium containing 34 μg/mL Cm and then cultured at 37°C with shaking at 180 rpm. When the OD_600_ of the bacterial culture reached 0.6-0.9, the cells were induced by IPTG at a final concentration of 0.25 mM for 24 h. Then, the off-gas samples were taken from the headspace of the sealed cultures and analyzed by GC-MS.

Products characterization was carried out by capillary GC-MS using an Agilent 5975C system chromatograph. A HP-INNOWAX capillary column (30 m × 0.25 mm × 0.25 μm, Agilent, Palo Alto, CA, USA) was used, with helium as the carrier gas at a flow rate of 1 ml min^-1^. The following oven temperature program was carried out: 40°C for 1 min, increase of 4°C/min to 70°C, then programmed from 70°C to 250°C at 25°C/min, where it was held for 5 min. The injector temperature was maintained at 250°C; ion source temperature 230°C; EI 70 eV; mass range 35-300 m/z. suitable amount of samples were injected in split injection mode with a 20:1 split ratio. Peak identification was based on the relative retention time and total ion mass spectral comparison with the external standard.

### Quantification of sabinene by gas chromatography (GC)

The different strains were inoculated in 50 ml of fermentation medium containing 34 μg/mL Cm and/or 100 μg/mL Amp and then cultured under the conditions mentioned above. Finally, the off-gas samples were taken from the headspace of the sealed cultures and analyzed by GC.

The GC analysis was performed on an Agilent 7890A equipped with a flame ionization detector (FID). The separation of sabinene was performed using an HP-INNOWAX column (25 m × 250 μm × 0.2 μm). The linear velocity was 1 ml/min with N_2_ as carrier gas. The oven temperature was initially held at 50°C for 1 min, increased at 5°C/min to 100°C to 250°C, and finally held at 250°C for 5 min. The temperatures of injector and detector were held at 250°C and 260°C, respectively. The peak area was converted into sabinene concentration in comparison with a standard curve plotted with a set of known concentrations of sabinene which was bought from Sigma-Aldrich.

### Optimization of fermentation medium and process

Optimization of fermentation medium was performed in shake-flask experiments in triplicate series of 600 ml sealed shake flasks containing 50 ml of fermentation medium incubated with the strain HB4. Amp (100 μg/mL) and Cm (34 μg/mL) were added when it was necessary. *E. coli* strains were cultured in the broth for initial production of sabinene and incubated in a gyratory shaker incubator at 37°C and 180 rpm. When the OD_600_ reached 0.6-0.9 [40], IPTG was added to a final concentration of 0.25 mM, and the culture was further incubated at 30°C for 24 h. Then, 1 ml of gas sample from the headspace of the sealed cultures was quantified as described previously [43]. Concentrations of synthesized sabinene were calculated by converting the GC peak area into milligrams of sabinene via a calibration curve.

#### Effect of organic nitrogen source

The shake-flask cultures were incubated in initial medium with different organic nitrogen sources (5 g/L): beef extract (solarbio), beef powder (MDBio, Inc), tryptone (Beijing AoBoXing Bio-Tech Co., Ltd) or yeast extract powder (Beijing AoBoXing Bio-Tech Co., Ltd)) at the above-mentioned culture conditions, and the sabinene products were detected.

#### Effect of carbon source

Carbon source is the main feedstock in fermentation. Therefore, the commonly used carbon sources (glucose and glycerol, 20 g/L) were screened in shake-flask with the nitrogen-optimized initial medium, at the above-mentioned culture conditions.

#### Effect of induction temperature

The *E. coli* strain was inoculated in 50 ml of optimized fermentation medium and cultured with shaking at 180 rpm. The shake-flask cultures were incubated at different induction temperatures (25°C, 28°C, 31°C, 34°C or 37°C), when the OD_600_ of the bacterial culture reached 0.6-0.9, for 24 h in a final concentration of 0.5 mM, and the sabinene products were quantified.

#### Effect of IPTG concentration

The shake-flask culture was incubated in different inducer (IPTG) concentrations (0.05 mM, 0.1 mM, 0.25 mM, 0.5 mM or 1 mM) at the optimized temperature for 24 h, and the sabinene products were measured.

#### Toxicity of commercial sabinene to E. coli

The sealed shake-flask culture was incubated in 50 ml of optimized fermentation medium and cultured with shaking at 180 rpm at a temperature of 31°C, with different concentration of commercial sabinene (0.5 g/L, 1 g/L, 2.5 g/L, and 5 g/L). Meanwhile, the growth of the bacterial culture was determined by measuring the OD_600_ (the optical density at 600 nm) with a spectrophotometer (Cary 50 UV-Vis, Varian) at 4 h, 7 h, 11 h, 14 h, 17 h, 20 h, 24 h and 36 h. The inhibition rate (IR) was calculated by the following equation:

$$\mathrm{IR}=\left(1-OD_{600s}/OD_{600C}\right)\times 100\%$$

Where IR = Inhibition rate (100%); *OD*_*600s*_ = OD_600_ of sample; *OD*_*600C*_ = OD_600_ of control at the same time as the sample.

### Fed-batch fermentation

The strain HB4 harboring pHB7 and pTrcLower was inoculated to 5 ml of LB medium (Amp 100 μg/mL, Cm 34 μg/mL, 37°C, 180 rpm), and then 100 ml fresh LB medium with corresponding antibiotics was inoculated with the 5 ml overnight cultures, which were used to inoculate a 5-L fermentor (BIOSTAT Bplus MO5L, Sartorius, Germany) containing 2 L of optimized fermentation medium (Amp 100 μg/mL, Cm 34 μg/mL). The temperature was maintained at 37°C firstly, and then 30°C after induced. The pH was maintained at 7.0 via automated addition of ammonia, and foam development was prohibited with 1% Antifoam 204. The stirring speed was first set at 400 rpm and then linked to the dissolved oxygen (DO) concentration to maintain a 20% saturation of DO, the flow velocity of air was 1.5 L/min. The expression of heterogenous genes for sabinene production was initiated at an OD_600_ of 11 by adding IPTG at a final concentration of 0.15 mM, and IPTG was supplemented every 8 h. During the course of fermentation, the 40% glycerol was fed at a rate 4 g/L/h. Then, sabinene accumulation was measured every 60 min by GC as described above. Meanwhile, the growth of the bacterial culture was determined by measuring the OD_600_ with the spectrophotometer, and the dry cell weight was calculated according to the coefficient (one OD_600_ unit corresponded to 0.43 g/L of dry cell weight).

The specific productivity was calculated by the following equation [44].

$$Q_{s}=\frac{s_{1}-s_{0}}{t_{1}-t_{0}}\times \frac{2}{x_{1}+x_{0}}$$

Where Q_s_ = specific production rate (g/h/g dry cells); s = sabinene concentration (g/L); t = cultivation time (h), and x = biomass (g/L).

Conversion efficiency (gram to gram) of glycerol to sabinene was calculated by the following equation:

$$Y=G_{s}/G_{g}\times 100\%$$

Where Y = conversion efficiency (gram to gram, 100%); *G*_*s*_ = weight of sabinene (g); *G*_*g*_ = weight of glycerol (g).

## Abbreviations

Amp: Ampicillin; Cm: Chloramphenicol; DMAPP: Dimethylallyl pyrophosphate; IPP: Isopentenyl pyrophosphate; GPP: Geranyl diphosphate; GPPS: Geranyl diphosphate synthase; MVA: Mevalonate; MEP: Methylerythritol 4-phosphate; IPTG: Isopropyl β-D-thiogalactoside; PCR: Polymerase chain reaction; GC: Gas chromatography; GC-MS: Gas chromatography-mass spectrography; DO: Dissolved oxygen.

## Competing interests

The authors declare that they have no competing interests.

## Authors’ contributions

MX and JY developed the idea for the study, and helped to revise the manuscript. HZ designed the research, did the literature review and prepared the manuscript. HZ, QL, and JY did experiments, plasmid construction, strain cultivation, Fed-Batch fermentation and product detection. All authors read and approved the final manuscript.
